# Design of a synthesis‐friendly hypoxia‐responsive promoter for cell‐based therapeutics

**DOI:** 10.1002/elsc.202100045

**Published:** 2021-10-29

**Authors:** Chee Ka Candice Lam, Kevin Truong

**Affiliations:** ^1^ Institute of Biomedical Engineering University of Toronto Toronto ON Canada; ^2^ Edward S. Rogers Sr. Department of Electrical and Computer Engineering University of Toronto Toronto ON Canada

**Keywords:** biosensor, hypoxia, inducible expression, promoter design, synthetic promoters

## Abstract

Towards the goal of making ‘smart’ cell therapies, one that recognizes disease conditions (e.g. hypoxia) and then produces mitigating biologics, it is important to develop suitable promoters. Currently, hypoxia responsive promoters are composed of strongly repeated sequences containing hypoxia response elements upstream of a minimal core promoter. Unfortunately, such repeated sequences have inherent genomic instability that may compromise the long‐term consistency of cell‐based therapeutics. Thus, we designed a synthesis‐friendly hypoxia‐inducible promoter (named SFHp) that has GC content between 25% and 75% and no repeats greater than 9 base pairs. In HEK293 cells stably integrated with genes regulated by synthetic SFHp, we demonstrated inducible reporter expression with fluorescent proteins, cell morphology rewiring with our previously engineered RhoA protein and intercellular cell signalling with secreted cytokines. These experiments exemplify the potential usage of SFHp in cell‐based therapeutics with integrated genetic circuits that inducibly respond to the disease microenvironment.

**Abbreviations**
HIF: hypoxia‐inducible transcription factor
HRE: hypoxia response element
SFHp: synthesis‐friendly hypoxia‐inducible promoter
TSS: transcriptional start site

## 1 INTRODUCTION

Cell‐based therapeutics use genetically engineered cells as vehicles of delivery for biologics in the treatment or prevention of diseases [1, 2]. In these therapies, the stable integration of transgenes, most commonly through lentiviral or retroviral methods, is critical for consistent clinical responses in patients [3]. While constitutive gene expression is most common, the creation of a ‘smart’ cell‐based therapy requires the cell sense disease conditions and then subsequently secrete the mitigating therapeutic protein. Such inducible expression is especially important due to their ability to reduce side‐effects within the cell such as death, delayed growth, compensatory adaptations and off‐target effects throughout the body [4]. Variations in spatial and temporal phenotype can be accomplished with regulated promoters that reversibly fine‐tune levels of expression in response to different environmental conditions based on the activation and inactivation of transcription factors. For example, studies demonstrate naturally occurring inducible systems activated by different environmental stressors such as hypoxia, temperature, nutrient‐availability and mechanical‐stimuli as mechanisms of controlling mammalian gene expression [5, 6, 7, 8]. In clinical settings, hypoxia‐induced gene regulation is particularly important to harness due to the wide‐spread presence of hypoxia in disease microenvironments [9].

Promoters regulate transcription initiation at the correct transcriptional start site (TSS) and control expression levels depending on the conditions of the cell. Both of these are achieved through the use of conserved sequence elements located upstream of transgenes. The minimal core promoter is responsible for specifying the TSS to transcription machinery [10]. It often contains the evolutionarily conserved TATA box, TATAWAWR and mammalian initiator sequence, YYANWYY, which encompasses the TSS [10, 11]. Alone, the core promoter has very low basal expression levels. Higher levels of expression require enhancer sequences that bind activating transcription factors adjacent to the promoter and in turn recruit transcription machinery [12]. Accordingly, inducible expression of genes is established by conditional binding of transcription factors to distal and/or proximal enhancer regions upon specific environmental stimuli, drastically increasing the relative transcription levels compared to the non‐induced minimal promoter alone. For hypoxia‐induced response in cells, this process is mediated by signalling pathways that control hypoxia‐inducible transcription factor (HIF)‐alpha subunit and HIF‐beta that dimerize and bind to hypoxia response elements (HREs) when localized to the nucleus [13, 14].

Targeting the hypoxic microenvironment with cell‐based therapeutics is particularly promising because of its role in a number of different diseases, including heart disease, cancer and rheumatoid arthritis [15, 16]. While it is possible to use endogenous promoters (e.g. the VEGF or EPO promoter) that have HRE sites, these promoters contain other response elements that may be inducible by other pathways [17, 18]. To avoid these cross pathway effects, it is possible to design custom promoters with only HRE sites. Since each transcription factor binding site functions as an independent unit, they can be combined in different manners to vary promoter expression levels as long as the minimal core promoter remains intact [19]. For example, the commonly used commercial hypoxia‐responsive promoters were designed to include several repeats of HRE (i.e. the conserved binding site for HIF dimers) placed upstream of a minimal core promoter [20, 21, 22]. Unfortunately, the sequence repeats in these promoters are considered complex for gene synthesis because of the interference of repeating sequences during Gibson assembly [23]. More concerning, these exact sequence repeats make the genetic material unstable due to a greater potential for replication slippage, misalignment and homologous recombination [24]. As a result of genomic instability arising from repeats, our group and others have designed promoter sequences to be less repetitive [25, 26]. Outside of repeats, high GC content (e.g. 80% is considered very high) is also undesired for gene synthesis because PCR itself becomes inefficient [27]. Thus, to enable both cost effective de novo synthesis and genomic stability, we have created a synthesis‐friendly alternative, named synthesis‐friendly hypoxia‐inducible promoter (SFHp), with no extreme GC content or repeats greater that 9 base pairs. To demonstrate the functionality of SFHp, we used an established chemical‐based model to activate the HIF signalling pathway [28, 29, 30]. These experiments showed our promoter can be induced to express proteins for fluorescence imaging, protein switches for pathway rewiring and cytokines for intercellular signalling. In particular, the use of SFHp to produce a cytokine or signalling molecule allows communication and coordination with other engineered cell lines or host cells for developing more versatile cell‐based therapies.

**PRACTICAL APPLICATION**

The SFH promoter can be used in cell‐based therapeutics to conditionally regulate the expression of therapeutic transgenes in hypoxic microenvironments. The lack of exact repeats in the SFH promoter should improve genomic stability and cell line consistency over long‐term culturing.

## 2 MATERIALS AND METHODS

### 2.1 Promoter design

HRE with the consensus sequence 5′ TACGTG 3′ was used to design the enhancer region of SFHp. The exact repeat including the HRE (i.e. 5′ GTGACTACGTGCTGCCTAG 3′) from plasmid pGL4.42 was concatenated seven times to form the seed sequence for optimization. This seed sequence was optimized using the dnachisel python package with constraints that enforce no repeats greater than 9 bp, GC content between 25% and 75% and seven occurrences of the HRE (i.e. 5′ TACGTG 3′). Upon program execution, dnachisel randomizes the seed sequence until all constraints are satisfied. The final optimized seed sequence was then concatenated with the minimal CMV core promoter to create SFHp. Sequence repeats were identified using the EMBOSS dottup online tool with a window size of 10 bp, plotting each sequence against itself. GC‐content plots were generated in RStudio using packages ‘seqinr’ and ‘ggplot2’ with a window size of 30 bp.

### 2.2 Plasmid construction

All synthesis and subcloning of plasmids was done by Genscript. The SFHp sequence is CGTGAATACGTGCCGCGTAGAGGACTCACACGTACGCGTCGCGAACTACGTGATGCCGAGCCGATCCTCACGTAGGCGGACGTGACTACGTGCTGCCTAGACTACCCGCACGTACGGAGACGTCACTACGTGTAGCCTATCGCGGTAGGCGTGTACGGTGGGAGGTCTATATAAGCAGAGCTCGTTTAGTGAACCGTCAGATCGCCTGGAGACGCCATCCACGCTGTTTTGACCTCCATAGAAGACACCGGGACCGATCCAGCAGGCCTGGATCCGCCGCCACCATG. The HRE promoter from plasmid pGL4.42 is CGTGACTACGTGCTGCCTAGCGTGACTACGTGCTGCCTAGCGTGACTACGTGCTGCCTAGCGTGACTACGTGCTGCCTAGCGCGGAGACACTAGAGGGTATATAATGGAAGCTCGACTTCCAGCTTGGCAATCCGGTACTGTTGGTAAAGCCGCCATG. Active tumour necrosis factor alpha (TNFα) was assembled as the tandem fusion of the IL4 leader sequence (^1^MGLTSQLLPPLFFLLACAGNFVHG^24^) and a fragment resulting from TACE cleavage of TNFα (amino acids 77–233). Similarly, the Ceru, CaRQ, Venus and active TNFα sequences were human codon‐optimized inserted into the SFHp plasmids by Genscript (Table S1).

### 2.3 Stable cell culture creation

Cells were maintained in Dulbecco's modified Eagle's medium containing 25 mM D‐glucose, 1 mM sodium pyruvate and 4 mM L‐glutamine (Invitrogen, Carlsbad, CA, USA) supplemented with 10% fetal bovine serum (Sigma‐Aldrich, St. Louis, MO, USA) in T25 flasks and incubated at 37°C and 5% CO_2_. The above designed plasmids were used to create stable HEK293 cells by lentiviral infection as previously described [31]. Specifically, HEK293 cells at 90% confluency were infected with lentivirus containing genetic cargo of interest for 24 h. The growth media was changed the next day and the cells were grown for 1 week to allow sufficient time for genomic integration. Then, the cells are serially diluted into 96‐wells with 1 μg/mL of puromycin resistance for selection. After 2 weeks, colonies derived from single cells were isolated from individual wells of the 96‐well plate. Individual colonies were screened for inducibility after a 24 h stimulation with 1 mM dimethyloxalylglycin (DMOG) (Cedarlane).

### 2.4 Imaging

Prior to imaging, cells were plated in 96‐well glass‐bottom plates (Mattek). Images were taken with the Olympus IX81 microscope, using a Lambda DG4 xenon lamp for the light source, and a QuantEM 512SC CCD camera with a 10× objective or 40× objective (Olympus). Excitation (EX) and emission (EM) filter bandpass specifications were as follows, for fluorescent proteins: Ceru (EX: 438/24, EM: 482/32) and Venus (EX: 500/24, EM: 524/27) (Semrock). Images were analysed via ImageJ and μManager software. Time‐lapse images were taken 10 s apart with 1 s exposure. Imaging was conducted with cells washed and maintained in PBS with CaCl_2_ (Sigma). Blebbing was induced in HEK293 cells expressing CaRQ with stimulation of 10 μM ATP (Sigma).

## 3 RESULTS AND DISCUSSION

### 3.1 Design of a SFHp

**FIGURE 1:**
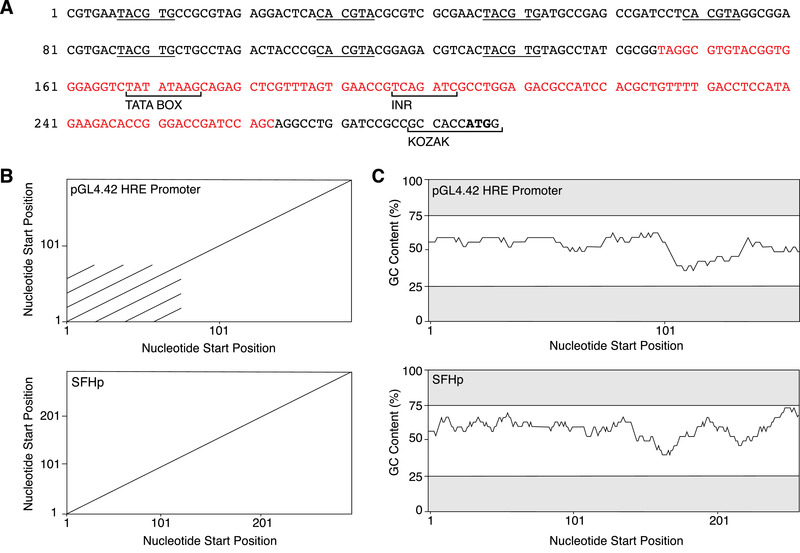
Promoter design. (A) Sequence of synthetic, synthesis‐friendly, hypoxia‐inducible promoter SFHp. Features are denoted by brackets and labelled underneath the sequence. Hypoxia response elements (HREs), the conserved HIF dimer binding site, have forward sequence of TACGTG and are underlined. There are seven HRE sites alternating in the sense and antisense strands. Sequence highlighted in red represents the minimal CMV promoter containing the TATA box and Inr consensus sequences. The Kozak sequence surrounds translation start codon ATG, in bold. (B) Dot plots represent repeating sequences in the forward direction for a reference hypoxia‐inducible promoter from plasmid pGL4.42 (Promega), top, and our SFHp sequence, bottom. Dot plot is created using a window size of 9 bp. (C) GC‐content plots for reference hypoxia‐inducible promoter, top, and SFHp, bottom, generated with window size of 30 bp. Thresholds of 25% and 75% are shown as limits for synthesis‐friendly GC nucleotide compositions. Mammalian initiator sequence (Inr)

An SFHp was created by staggering the placement of seven HRE (i.e. 5′ TACGTG 3′) that have no repeats in the flanking subsequences upstream of a minimal CMV core promoter [32]. The HRE sites allow hypoxia dependent binding by HIF dimers and subsequent gene expression while the core promoter allows accurate initiation at the TSS. Using self dot plots to identify repeated sequences (i.e. diagonal lines on the dot plot), we detected that the commonly used commercial HRE promoter from plasmid pGL4.42 (Promega) has strongly repeated segments arising from the four HRE and flanking subsequences that are exactly the same (Figure 1B, top). In contrast, our SFHp lacks any strongly repeated segments by eliminating all repeats greater than 9 base pairs in the forward or reverse direction (Figure 1B, bottom). This was accomplished by varying spacing and randomizing bases flanking the HRE sites. Our final design of SFHp contained seven HRE sequences compared to the four HRE sequences found in pGL4.42 (Figure 1A). Previous studies with HRE‐based promoters have shown that hypoxia inducibility is dramatically increased with only two HRE sites but there is further inducibility with more HRE sites although less and less improvement was observed [33]. As we altered the flanking subsequences, we also maintained the GC content of each 30 bp window of the promoter within the synthesis friendly range of 25–75% (Figure 1C).

### 3.2 SFHp can be induced to express fluorescent protein

**FIGURE 2:**
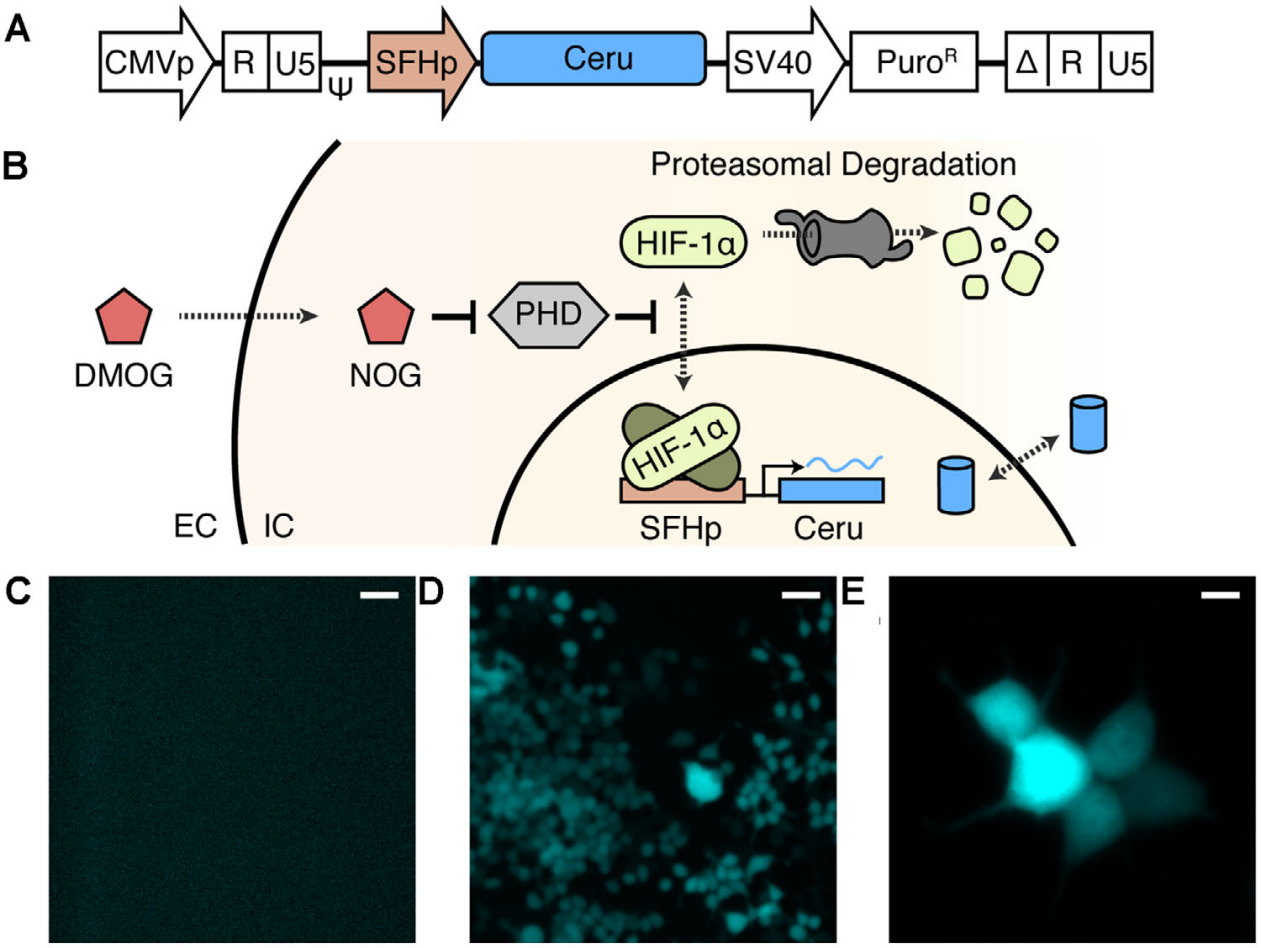
SFHp‐directed expression of fluorescence marker. (A) Schematic of transfer vector for lentiviral infection of HEK293 cells for Cerulean (Ceru) expression. (B) Cartoon of DMOG stimulation of HIF‐1α transcription factor to activate expression from HRE‐containing promoters, notably, SFHp. DMOG added in cell culture permeates cell and is transformed into NOG that inhibits PHD's destabilizing activity on HIF‐1α by targeting HIF‐1α for proteasomal degradation. Fluorescent protein Cerulean is able to passively diffuse between cytosolic space and nucleus. Pseudo‐coloured fluorescence microscopy images of stably transfected HEK293 cells expressing construct from (A) before DMOG stimulation at 10× (C), 24 h after 1 mM DMOG addition at 10× (D) and 40× magnification (E). Scale bar represents 40 μm for 10× images and 10 μm for 40× images. The results were consistent in three independent experiments with at least 100 cells or 10 cells in the field of view of each image at 10× or 40× magnification, respectively. DMOG, dimethyloxalylglycine; EC, extracellular space; NOG, *N*‐oxylglycine; PHDs, prolyl hydroxylase domain proteins

To show that SFHp has inducible gene expression, we created stable HEK293 cells by lentiviral infection that expressed cyan fluorescent protein Cerulean (hereafter, Ceru) all under regulation of SFHp (Figure 2A). HEK293 cell line was chosen for all our tests because it easily uptakes genetic material [34] and has also been frequently used to test hypoxia inducible promoters. Previously, HEK293 cells have been used in hypoxia specific gene therapy studies for diseases such as breast cancer tumours, ischemic myocardium and sleep apnoea [35, 36, 37]. For reproducible and robust results, we used lentiviral infections to create stable integrations of the above construct into HEK293 cells and subsequent serial dilution to select cells growing from a single progenitor cell to ensure consistent cell‐wide Ceru expression upon hypoxic conditions (Figure 2C–E). Ceru expression was stimulated by the addition of 1 mM DMOG into the cell culture media. This is an established model used to express genes that respond to hypoxia through induction of HIF‐1α signal pathway, despite normoxic cell culture environment [28, 29, 30]. From the media, DMOG readily permeates the extracellular membrane and is converted by de‐esterization to *N*‐oxylglycine (NOG) in the cytosol. NOG functions as a competitive inhibitor of prolyl hydroxylase domain proteins and blocks their ability to attach hydroxylase modifications on HIF‐1α transcription factors. These modifications occur during normoxic conditions to label HIF‐1α for proteasome degradation. In the absence of hydroxylation, cytoplasmic HIF‐1α concentration is stabilized and translocated across the nuclear envelope where HIF‐1α dimerizes with HIF‐1β to bind and activate gene transcription from promoters containing HRE sites [38]. Thus, addition of DMOG results in hypoxic gene expression profiles expected for HRE promoters like our synthetic SFHp. To test that SFHp behaved similarly to the commercial HRE promoter in pGL4.42, transient transfection experiments were performed in HEK293 cells upon induction with DMOG (Figure S2). Unfortunately, the generation of stable cells using the commercial HRE promoter in pGL4.42 was challenging, possibly due the presence of the exact repeats causing slippage. Next, we imaged the SFHp stable cells at 10× and 40× magnification, 24 h after 1 mM DMOG addition, and clearly observed expression of Ceru fluorescent protein in every HEK293 cell (Figure 2D,E). The absence of fluorescence before DMOG stimulation indicates that our synthetic SFHp promoter is inducible in response to HIF‐1α activity (Figure 2C). Furthermore, by nature of our single progenitor cell selection process following integration, the activity of our inducible SFHp promoter was reversible upon removable of DMOG from the media.

### 3.3 SFHp can express functional membrane‐localized engineered RhoA

**FIGURE 3:**
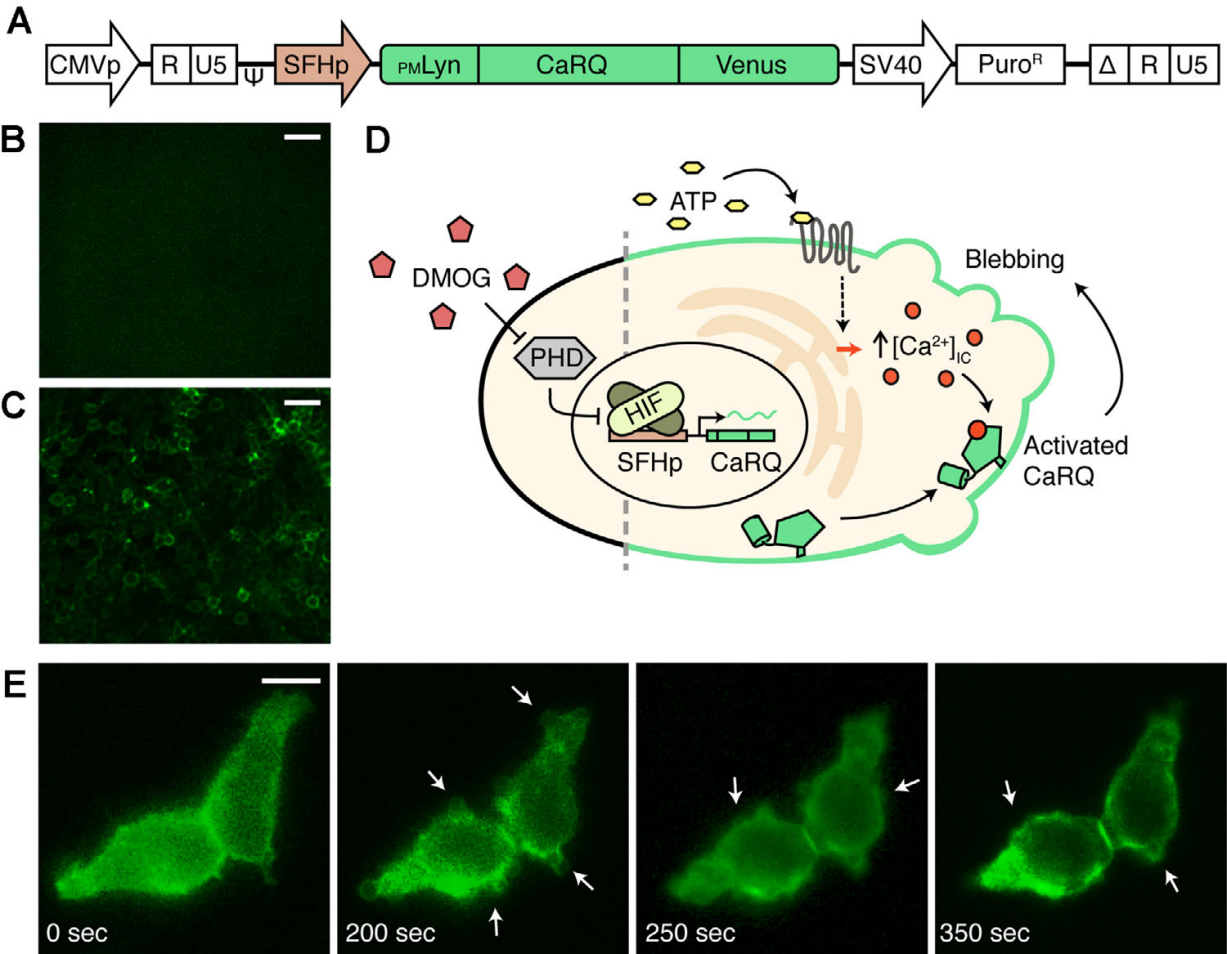
SFHp‐directed expression of functional CaRQ. (A) Schematic of lentiviral transfer vector for HEK293 infection. Engineered Ca^2+^‐responsive RhoA (CaRQ) is fused to plasma localization tag _PM_Lyn at the N‐terminus and Venus fluorescent protein at the C‐terminus. 10× magnification fluorescent microscopy images of transfected HEK293 cells before (B) and 24 h after (C) 1 mM addition of DMOG into cell culture media, scale bar represents 30 μm. (D) Cartoon of DMOG stimulation of SFHp expression and ATP stimulation of Ca^2+^‐dependent blebbing activity of membrane‐localized CaRQ. (E) Images of transfected and DMOG‐stimluated HEK293 cells taken from time‐lapse at 40× magnification following bolus addition of 10 μM ATP at 0 s. The results were consistent in three independent experiments with at least 100 cells or five cells in the field of view of each image at 10× or 40× magnification, respectively. Arrows point to changes in cell membrane projections and blebbing. Scale bar represents 10 μm

To show that SFHp can inducibly express proteins that can alter the intracellular functions, we created stable HEK293 cells by lentiviral infection that expressed an engineered Ca^2+^ activated RhoA protein (named CaRQ) under SFHp (Figure 3A). Our group previously engineered CaRQ as a chimeric RhoA GTPase that requires binding to Ca^2+^ in order to fold into the active structure for cytoskeletal remodelling (e.g. blebbing) ultimately resulting in directed cell migration (Figure 3D). The Ca^2+^ sensitivity of CaRQ allows cell migration to be rewired to any external stimulus that produces a cytosolic Ca^2+^ signal [39]. To direct plasma membrane localization, CaRQ is fused to the Lyn sequence MGCIKSKGKDSA (called _PM_Lyn) at the N‐terminus; to visualize expression, it is fused to the yellow fluorescent protein Venus [40]. Likewise, for reproducible and robust results, we used lentiviral infections to create stable integrations of the above construct into HEK293 cells and subsequent serial dilution to select cells arising from a single progenitor. As expected, there was Venus fluorescence at the plasma membrane of these cells following addition of DMOG stimulus (Figure 3B,C). Since _PM_Lyn requires Met1 to be cleaved for lipid modifications that anchor tagged the protein to the plasma membrane, the apparent membrane localization of CaRQ means transcription occurred at the SFH promoter rather than by read‐through expression [41]. Next, a bolus addition of 10 μM ATP was used to trigger Ca^2+^ efflux from endoplasmic reticulum, thereby raising cytosolic Ca^2+^ concentrations [39]. After a couple minutes, we observed the formation and retraction of blebs and projections at the cell plasma membrane indicating activation of CaRQ migratory activity (Figure 3E) (Video S1). Since the expression under SFHp retained functionality of the engineered protein CaRQ, a ‘smart’ cell‐based therapy could potentially be engineered with SFHp such that it recognizes a hypoxia environment and expresses a multi‐component gene circuit that rewires cell migration to seek local pathogens. This would allow the engineering of multiple conditions to be satisfied before any therapeutic intervention, providing greater specificity and reduce toxicity. For instance, hypoxia is common in the microenvironment of the tissue surrounding a bacterial infection [42]. A CaRQ‐based migration circuit and lysozyme could be regulated by SFHp to seek lipopolysaccharides secreted by bacteria to administer lysozyme locally. If the seeking circuit was regulated under a constitutive promoter instead, it might unintentionally treat ‘good’ bacteria in the gut.

### 3.4 SFHp can express functional TNFα for intercellular signalling

**FIGURE 4:**
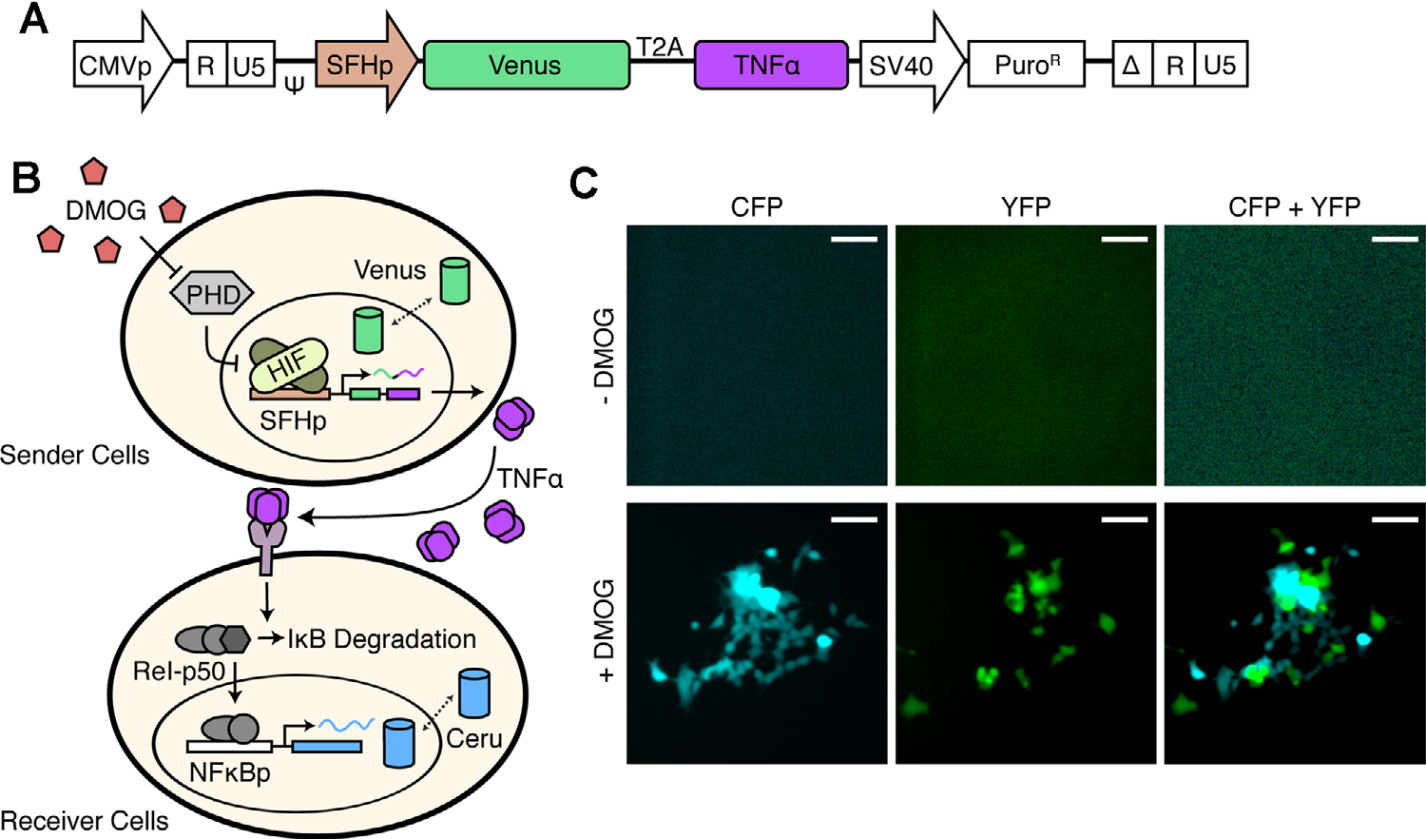
SFHp‐directed expression of bicistronic sequence in sender cell for intercellular signalling. (A) Schematic of lentiviral transfer vector containing synthetic SFHp controlling expression of Venus fluorescent protein and active TNFα as a bicistronic sequence separated by co‐translational cleavage site T2A. (B) Cartoon of co‐culture communication between sender (top) and receiver (bottom) cells. DMOG stimulation of SFHp construct results in Venus expression in sender cells and TNFα secretion. TNFα binds receiver cell receptor that signals for degradation of inhibitory protein IκB, freeing NFκB transcription factor subunits Rel and p50 to translocate into nucleus and activate NFκB promoters. (C) Microscopy imaging of co‐culture plate at 10× magnification before DMOG stimulation for Cerulean fluorescence and Venus fluorescence, and 24 h after DMOG stimulation. The results were consistent in three independent experiments with at least 100 cells in the field of view of each image. Scale bar represents 40 μm. T2A, *Thosea asigna* virus 2A site; TNFα, tumour necrosis factor alpha

To show that SFHp can inducibly express paracrine signals for the intercellular signalling, we created stable HEK293 cells (called ‘sender’ cells) by lentiviral infection that expressed Venus and active TNFα signalling protein joined in tandem by a T2A co‐translational, ‘self‐cleavage’ site—all regulated by SFHp (Figure 4A). The T2A sequence was derived from *Thosea asigna* virus and shown to have high cleavage efficiency allowing for expression of bicistronic sequence from a single promoter [43]. As expected, we observed cell‐wide Venus fluorescence 24 h after addition of DMOG (Figure 4C). Similarly, the stable HEK293 cells called ‘receiver’ cells were created by lentiviral infection that expressed Ceru under the regulation of a promoter containing NF‐kB response elements derived from the commonly used commercial NF‐kB promoter from plasmid pGL4.32 (Promega). This promoter was not optimized to be synthesis‐friendly. As expected, receiver cells express Ceru throughout the cell 24 h after induction by addition of 10 ng/mL of active TNFα (Figure S1). When the sender and receiver cell lines were co‐incubated together and then induced by addition of DMOG for 24 h, the different cell lines fluoresced Venus and Ceru, respectively (Figure 4C). This sense‐response system demonstrates the use of SFHp for inducible intercellular communication with other engineered cell lines. Potentially in cell‐based therapies, it allows us to pair the sender cells with a variety of different universal receiver cells responding to sender cell's TNFα signal. These therapeutic receiver cell lines can likewise be paired with different universal sender cell lines that secrete TNFα under the regulation of other inducible promoters that respond to different stimulus or environmental conditions. Through this kind of rewiring of intercellular signalling, fewer specific cell lines are need to be engineered to have a variety of re‐configurable effects. For instance, with a sender cell that drives TNFα expression under SFHp and with a library of receiver cells that drive the TNFα‐inducible expression of target genes such as lysozyme (for bactericide), or cathepsin G (for bactericide) or CaRQ‐based circuit (for directed migration), it is possible to re‐configure the hxpoxia‐inducible expression of any combination of target genes to test treatment schemas using intercellular signalling. As the library of sender cells expands, the target gene expression can be rewired via intercellular signalling to other inducible stimuli beyond hypoxia.

## 4 CONCLUDING REMARKS

We engineered a synthesis‐friendly promoter named SFHp that constrains gene expression to hypoxic conditions when stably integrated into HEK293 cells. To do so, we arranged hypoxia responsive elements upstream of a minimal CMV core promoter and checked for repeating sequences and suitable GC content. These two features are common culprits that complicate the synthesis of engineered sequences and subject them to higher prices when commercially synthesized. Furthermore, the lack of these repeated subsequences in SFHp should make the genetic material more stable and less likely corrupted when integrated into ‘smart’ cell‐based therapeutics. We demonstrated the ability of SFHp to express fluorescent proteins (i.e. Ceru and Venus) and engineered protein switches (i.e. CaRQ) upon addition of the DMOG chemical used to activate the HIF‐1α transcription factor. Furthermore, we used SFHp‐controlled expression of active TNFα, called sender cells, to act as a paracrine signal for hypoxic conditions. This intercellular communication was demonstrated by co‐culturing sender cells with receiver cells containing a NFκB‐regulated gene circuit. We envision that our synthetic promoter SFHp can be used in engineered therapeutic cells that are administered systemically in order to identify and conditionally express active genes at hypoxic environments within the human body. Hypoxia can act as a therapeutic target for a number of different diseases, including heart disease, cancer and rheumatoid arthritis [15, 16]. As synthetic biology and cellular engineering continues to evolve, it will be important to incorporate systems that exploit the living cell's ability to detect spatial cues and regulate expression accordingly. The development of our SFHp should allow for the stable integration of genetic material into cell‐based therapeutics to detect the hypoxia microenvironment and only then produce therapeutic genes (e.g. erythropoietin).

## CONFLICT OF INTEREST

The authors have declared no conflict of interest.

## Supporting information

Supporting information

Supporting information

## Data Availability

All data generated or analysed during this study are included in this published article (and its supplementary information files).
