# Supplementary material for: Design of a synthesis‐friendly hypoxia‐responsive promoter for cell‐based therapeutics
Source: Eng Life Sci. 2021 Oct 29;21(12):848–56. doi: 10.1002/elsc.202100045 (PMC8638314; doi:10.1002/elsc.202100045)
Supplement: Supplementary file 1 — Supporting information [file ELSC-21-848-s001.docx]

**Engineering a synthesis-friendly hypoxia-inducible promoter for mammalian cell expression**

Chee Ka Candice Lam^1^ and Kevin Truong^1,2,*^

^1^ Institute of Biomedical Engineering, University of Toronto, 164 College Street, Toronto, Ontario, M5S 3G9, Canada

^2^ Edward S. Rogers, Sr. Department of Electrical and Computer Engineering, University of Toronto, 10 King’s College Circle, Toronto, Ontario, M5S 3G4, Canada

Lead contact (*):

Kevin Truong

[kevin.truong@utoronto.ca](mailto:kevin.truong@utoronto.ca), Tel: 416-978-7772, Fax: 416-978-4317

164 College Street Room 407, Rosebrugh Building, University of Toronto

Toronto, ON, M5S3G9, Canada

**Supporting Information**

**Table of Contents**

**Supplemental Table 1.** Sequences of proteins used in the study

**Supplemental Figure 1.** TNFα-inducible expression of fluorescent proteins in NFkB cell lines

**Supplemental Figure 2.** HEK293 transiently transfected with plasmids encoding HRE promoters

**Supplementary Video 1.** Fluorescence microscopy timelapse of HEK293 cells stably transfected with SHREp-driven CaRQ and Venus fusion construct.

**Supplemental Table 1**. Sequences of proteins used in the study

| **Protein** | **Sequence** |
| --- | --- |
| Cerulean | MVSKGEELFTGVVPILVELDGDVNGHKFSVSGEGEGDATYGKLTLKFICTTGKLPVPWPTLVTTLSWGVQCFARYPDHMKQHDFFKSAMPEGYVQERTIFFKDDGNYKTRAEVKFEGDTLVNRIELKGIDFKEDGNILGHKLEYNAIHGNVYITADKQKNGIKANFGLNCNIEDGSVQLADHYQQNTPIGDGPVLLPDNHYLSTQSKLSKDPNEKRDHMVLLEFVTAAGITLGMDELYK |
| CaRQ | MGCIKSKGKDSATSEQIAEFKEAFSLFDKDGDGTITTKELGTVMRSLGQNPTEAELQDMINEVDADGNGTIYFPEFLTMMARKMKDTDSEEEIREAFRVFDKDGNGYISAAELRHVMTNLGEKLTDEEVDEMIREADIDGDGQVNYEEFVQMMTAKASKRRWKKNFIAVSAANRYKKISSSGALASAAIRKKLVIVGDGACGKTCLLIVFSKDQFPEVYVPTVFENYVADIEVDASGSAITVQRYVRGIQARAYARFLASGKQVELALWDTAGLEDYDRLRPLSYPDTDVILMCFSIDSPDSLENIPEKWTPEVKHFCPNVPIILVGNKKDLRNDEHTRRELAKMKQEPVKPEEGRDMANRIGAFGYMECSAKTKDGVREVFEMATRAALQA |
| Venus | MVSKGEELFTGVVPILVELDGDVNGHKFSVSGEGEGDATYGKLTLKLICTTGKLPVPWPTLVTTLGYGLQCFARYPDHMKQHDFFKSAMPEGYVQERTIFFKDDGNYKTRAEVKFEGDTLVNRIELKGIDFKEDGNILGHKLEYNYNSHNVYITADKQKNGIKANFKIRHNIEDGGVQLADHYQQNTPIGDGPVLLPDNHYLSYQSKLSKDPNEKRDHMVLLEFVTAAGITLGMDELYK |
| active TNFα | MGLTSQLLPPLFFLLACAGNFVHGTSVRSSSRTPSDKPVAHVVANPQAEGQLQWLNRRANALLANGVELRDNQLVVPSEGLYLIYSQVLFKGQGCPSTHVLLTHTISRIAVSYQTKVNLLSAIKSPCQRETPEGAEAKPWYEPIYLGGVFQLEKGDRLSAEINRPDYLDFAESGQVYFGIIAL |


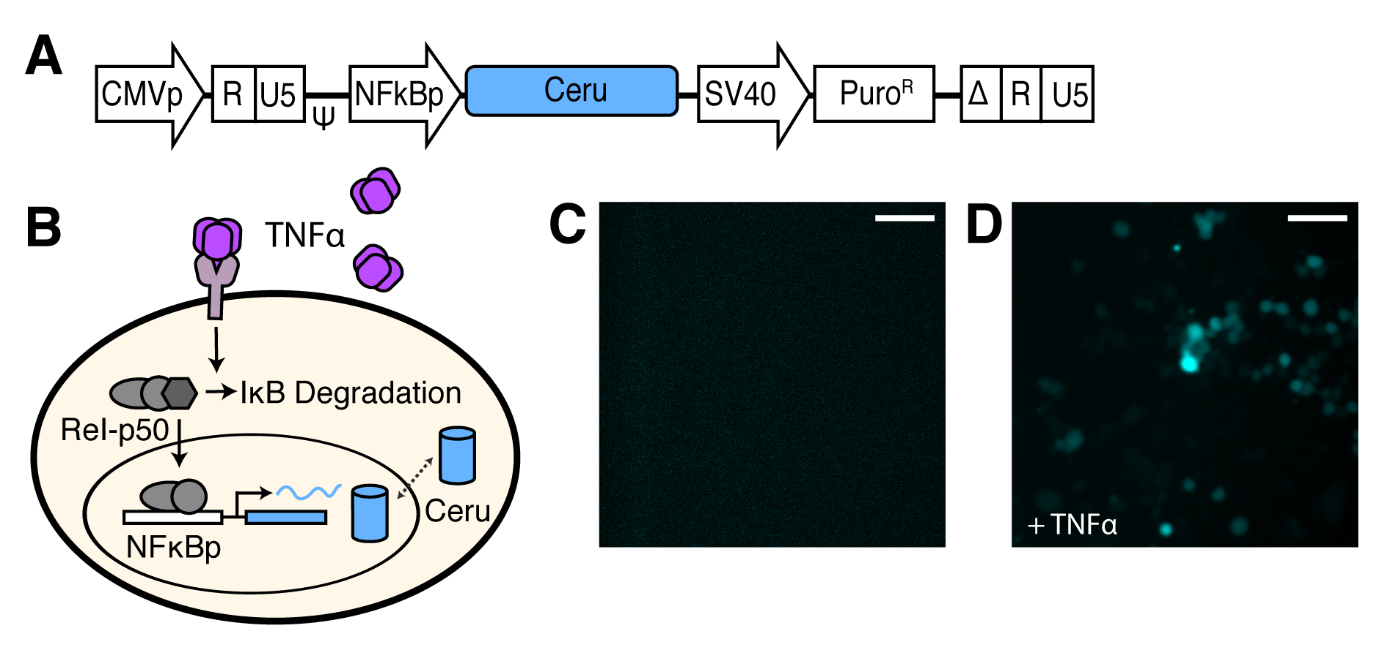


**Figure S1.** HEK293 receiver cells infected with lentiviral construct for TNFα detection. (A) Schematic of DNA construct for stable integration has Cerulean fluorescent protein under control of NFκB promoter. (B) Cartoon of TNFα binding endogenous HEK293 TNF receptors. Activated receptors trigger degradation of inhibiter protein IκB and allows NFκB subunits ReI-p50 to translocate into the nucleus to activate promoter expression. Fluorescence microscopy images for Ceru before (C) and 24 hours after TNFα addition to cell media (D). Scale bar represents 40μm. The results were consistent in 3 independent experiments with at least 100 cells in the field of view of each image.

**­­­
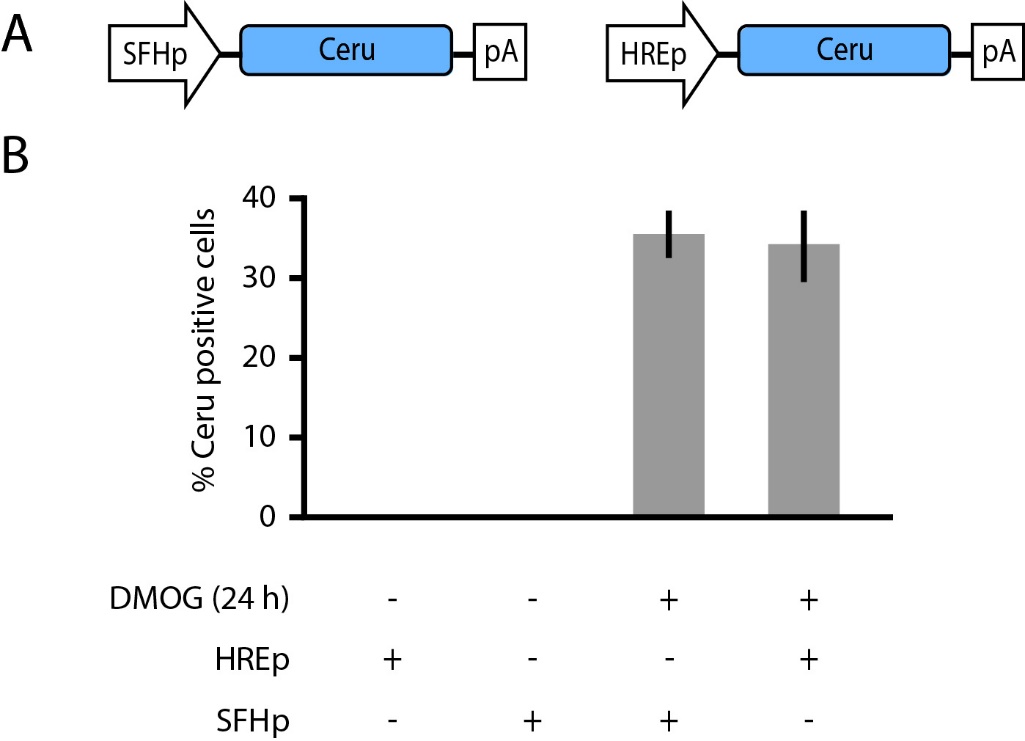
**

**Figure S2.** HEK293 transiently transfected with plasmids encoding the SFHp or the HRE promoter from the pGL4.42 plasmid (labelled HREp). (A) Schematic of DNA construct for transient transfection of SFHp or HREp driving expression of Cerulean cyan fluorescent protein. pA is the SV40 polyadenylation sequence. (B) Graph of the percentage of cyan fluorescent cells before and after 24 h treatment with 1 mM DMOG. Error bars (SD) are derived from 3 independent transfections with at least 100 cells in the field of view.

**Supplementary Video 1.** Fluorescence microscopy timelapse of HEK293 cells stably transfected with SFHp-driven CaRQ and Venus fusion construct described in Figure 3A. Images were taken at 10 second intervals with observation period to ensure absence of basal cell blebbing. Bolus 10uM ATP stimulus was added at 3 minutes 10 seconds. Rapid blebbing began at 6 minutes where cell membrane displayed multiple protrusions extending and contracting.
